# Gastric mucosa-associated lymphoid tissue lymphoma and *Helicobacter pylori* infection: a review of current diagnosis and management

**DOI:** 10.1186/s40364-016-0068-1

**Published:** 2016-07-27

**Authors:** Qinglong Hu, Yizhuo Zhang, Xiaoyan Zhang, Kai Fu

**Affiliations:** 1 Tucson Pathology Associates, PC Carondelet Saint Joseph Hospital, 351 North Wilmot Road, Tucson, AZ 85711 USA; 2 Department of Hematology, Tianjin Medical University Cancer Institute and Hospital, National Clinical Research Center of Cancer, Tianjin Key Laboratory of Cancer Prevention and Therapy, Tianjin, 300060 China; 3 Department of Pathology and Microbiology, University of Nebraska Medical Center, 983135 Nebraska Medical Center, Omaha, NE 68198 USA

**Keywords:** Gastric MALT, *Helicobacter pylori*

## Abstract

*Helicobacter pylori* (*H. pylori*)-associated gastritis is one of the most common infectious diseases in the United States, China and worldwide. Gastric mucosa-associated tissue lymphoma (MALT lymphoma) is a rare mature B-cell neoplasm associated with *H. pylori* infection that is curable by antibiotics therapy alone. The pathological diagnosis of gastric MALT lymphoma can be reached by histological examination, immunohistochemical staining and B-cell clonality analysis. *H. pylori* eradication is the choice of therapy for early-stage gastric MALT lymphoma. High response rates and long-term survival have been reported in refractory and localized diseases treated with low-dose radiation therapy. Systemic chemotherapy is recommended for advanced-stage gastric MALT lymphoma and cases with large B-cell lymphoma transformation. Recent advances in the pathological diagnosis and management of gastric MALT lymphoma are reviewed in this article.

## Background

*Helicobacter pylori* (*H. pylori*) infection of the stomach is one of the most common diseases worldwide. According to the World Health Organization, *H. pylori* infection contributes to approximately 75 % of stomach cancers and 5.5 % of all cancers worldwide [1]. The infection rate varies in different countries of the world and also in different regions of China, from 55 to 80 % in the mainland to 15 % in Hong Kong and 40 % in Taiwan [2–4]. The infection rate is higher in rural areas than in cities [4]. Gastric mucosa-associated lymphoid tissue (MALT) lymphoma is a clonal B-cell neoplasm arising from post-germinal center B-cells in the marginal zone of the lymphoid follicles*. H. pylori* was identified from the gastric mucosa of patients with active chronic gastritis more than 30 years ago by Marshall and Warren [5]. *H. pylori* infection of the stomach is considered a major cause of chronic active gastritis and a major risk factor for gastric MALT lymphoma. Since the discovery of the association of *H. pylori* gastritis with gastric MALT lymphoma [6], extensive basic studies and clinical trials have been performed worldwide, and treatment guidelines have been recommended for *H. pylori*-associated gastritis and gastric MALT lymphoma [7–11]. Gastric MALT lymphoma is a rare disease. The estimated incidence of gastric lymphoma was approximately 0.3–0.8 per 100,000 people in Europe [8]. The incidence of gastric MALT lymphoma was approximately 0.38 per 100,000 people in the United States, according to a recent study. The incidence rates increased with age [12]. No population-based studies have been reported in the Chinese literature, and the incidence of gastric MALT lymphoma is unknown in China. We searched literature of studies of *H. pylori* gastritis and gastric MALT lymphoma in English and Chinese and reviewed the recent advances in the diagnosis and management of these two closely related diseases. Current issues in the diagnosis and management of the disease are also discussed.

## Diagnosis of gastric MALT lymphoma

The diagnosis of gastric MALT lymphoma relies on clinical symptoms, endoscopic features and pathohistological examination of gastric biopsy tissue, as well as noninvasive tests for *H. pylori* infection, such as the ^13^C-urea breath test and the monoclonal stool antigen test. The clinical presentation of patients with gastric MALT lymphoma is nonspecific. The symptoms include dyspepsia, vague epigastric pain, bloating and heartburn; more severe and alarming symptoms are anemia, melena, hematemesis, vomiting and weight loss.

Endoscopic examination with biopsy and histopathologic examination is a standard practice in the diagnosis of gastric MALT lymphoma in the United States and the Western world. The macroscopic changes of the gastric mucosa in MALT lymphoma are nonspecific, including thickening of the mucosal folds, irregular nodules, polypoid lesions, petechiae, edema, erosion and ulcers. The distribution of the lesions is usually patchy, with multiple foci. Therefore, sampling at different anatomic sites is recommended during gastric endoscopic examination and biopsy. Biopsies should be taken from abnormal and normal areas, including the antrum, the greater and lesser curvatures, and the fundus. At least two biopsies should be taken from each site of the stomach, and the tissue from each biopsy site should be fixed in separate containers with 10 % buffered formalin [13]. Sampling from multiple sites may be difficult in practice as it increases risk of complications, such as acute gastric bleeding, especially in patients on anticoagulant therapy. *H. pylori*-associated chronic active gastritis is present in the majority of patients with gastric MALT lymphoma.

## Histopathologic features of gastric MALT lymphoma

Under microscopic examination, the biopsy tissue usually shows dense lymphoid infiltration with monotonous features. Germinal centers may be observed with the expansion of the marginal zone. The lymphoma cells are typically small and monotonous with monocytoid features, with small, round nuclei and a scant to moderate amount of pink to clear cytoplasm. Some cases may show prominent plasmacytoid differentiation mixed with small lymphocytes. The lymphocytes may infiltrate the glandular epithelium, resulting in the destruction of the glands, forming so-called “lymphoepithelial lesions”. The lymphocytes in the glands are B-cells, which may be identified by immunohistochemical staining for B-cell and epithelial markers such as CD20 and pan-cytokeratin. However, these stains are not used routinely. Common immunohistochemical stains may include the B-cell markers CD20 and CD79a and the T-cell markers CD3 and CD5. Staining for CD21 may be helpful to highlight residual lymphoid follicles and expanding marginal zones. Aberrant expression of CD43 by the B-cells may be identified, which is a supporting evidence for gastric MALT lymphoma. The diagnosis of gastric lymphoma can be established by morphologic examination and immunohistochemical evaluation. However, a majority of the cases may require clonality analysis of the B-cells by in situ hybridization for kappa and lambda light chains with formalin-fixed tissue sections. The expression of kappa/lambda light chain may be weak, and they often cannot be detected by in situ hybridization. Negative expression of the light chains cannot rule out the diagnosis of lymphoma. If the result of in situ hybridization is inconclusive, further molecular studies, such as polymerase chain reaction (PCR), for immunoglobulin heavy chain (IgH) gene rearrangement may be beneficial. A definitive diagnosis of gastric MALT lymphoma is supported if clonal IgH gene rearrangement is demonstrated by a PCR study. However, this result is not a prerequisite for the diagnosis of gastric MALT lymphoma [14]. A report of “atypical lymphoid proliferation, suspicious for MALT lymphoma” may be issued for ambiguous cases. Clinical follow-up and repeat biopsy may finally lead to a definitive diagnosis. Wotherspoon and colleagues [15] observed a spectrum of histological changes between normal gastric mucosa, chronic active gastritis and gastric MALT lymphoma, as shown in Table 1. This histological grading is helpful in differential diagnosis. Histopathologic changes of Types 3 and 4 requires immunohistologic staining and/or molecular investigation to reach a definitive diagnosis. The biopsy tissues taken by endoscopy are usually small, and the layer of muscularis propria may not be observed in most of the biopsies. The depth of invasion of the gastric wall by the lymphoma cells is important for disease staging, and it should be evaluated and documented if identified under a microscope. Monotonous and dense lymphoid infiltration in the gastric mucosa may raise the suspicion of gastric MALT lymphoma. This requires the pathologist’s experience and may be affected by the adequacy of tissue processing, the thickness of the tissue sectioning, and the quality of histologic staining. Immunohistochemical staining may show expansion of the B-cell compartment with sheets of B-cells. The thickness of the tissue sections can significantly affect the interpretation of compartment expansion of B-cells or T-cells. The tissue sections of the gastric biopsy should not be more than 3 μm in thickness. A false impression of mixed B-cell and T-cell proliferation may be obtained due to inadequate thickness of the tissue sections during immunostaining.

**Table 1:** Wotherspoon scale of confidence of histological diagnosis of lymphoma [15]

Grade	Description	Histological features
0	Normal	Scattered plasma cells in the lamina propria. No lymphoid follicles.
1	Chronic active gastritis	Small clusters of lymphocytes in the lamina propria. No lymphoid follicles. No LELs.
2	Chronic active gastritis with florid lymphoid follicle formation	Prominent lymphoid follicles with surrounding mantle zone and plasma cells.
3	Suspicious lymphoid infiltrate, probably reactive	Lymphoid follicles surrounded by CCL cells that infiltrate diffusely into the lamina propria and occasionally into the epithelium.
4	Suspicious lymphoid infiltrate, probably lymphoma	Lymphoid follicles surrounded by CCL cells that infiltrate diffusely into the lamina propria and into the epithelium in small groups.
5	Low-grade B-cell lymphoma of MALT	Presence of dense diffuse infiltrate of CCL cells in the lamina propria with prominent lymphoepithelial lesions.

*CCL cells* centrocyte-like cells (or marginal zone cells), *LEL* lymphoepithelial lesion

Gastric MALT lymphoma is a type of low-grade B-cell lymphoma with tissue infiltration by small lymphocytes. High-grade transformation to large B-cell lymphoma is uncommon. If high-grade lymphoma is present, the infiltration of large lymphoma cells may be a predominant component or a minor component depending on the stage of transformation. Identification of residual low-grade components of MALT lymphoma may be helpful for the differential diagnosis of large B-cell lymphoma transformed from gastric MALT lymphoma versus primary diffuse large B-cell lymphoma or secondary stomach involvement by diffuse large B-cell lymphoma arising from other primary organs. Focal large B-cell transformation of gastric MALT lymphoma is an unfavorable prognostic feature and should be documented in the pathology report when it presents [16–19].

**Fig. 1:**
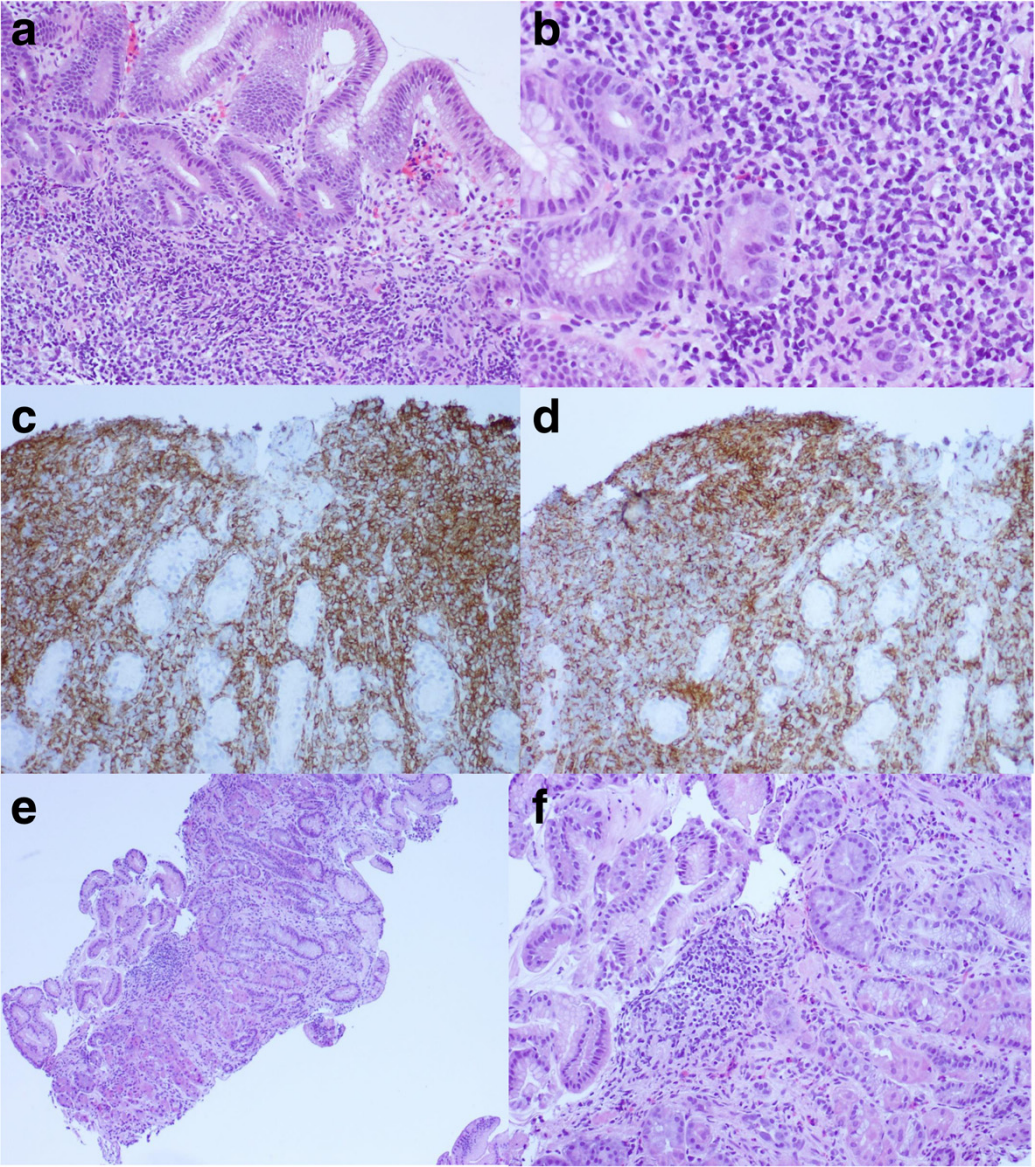
Gastric biopsy showing intra-lamina small lymphocyte infiltrate with monotonous features (**a**, original magnification 100 X); foci of lymphoepithelial lesion (**b**, H&E, original magnification 200 X); CD20 immunostaining shows sheets of CD20+ small B-cells (**c**, original magnification 200 X); aberrant expression of CD43 is also detected (**d**, original magnification 200X). A repeat gastric biopsy 6 months after *H. pylori* eradication showed scattered intra-lamina infiltrates of small lymphocytes, plasma cells and a few small loose lymphoid aggregates, indicating complete remission (**e**, H&E, original magnification 100 X; **f**, 200 X)

The majority of gastric MALT lymphoma is associated with *H. pylori* infection. *H. pylori* were reported in 50–100 % of Chinese patients with gastric MALT lymphoma [20–22]. Similar observations were also reported in other areas of the world [23]. Searching for *H. pylori* is recommended in routine H&E slides of gastric biopsies, and the status of *H. pylori* should be documented in the pathology report. Immunostaining for *H. pylori* on tissue sections appears to be the most specific and sensitive and should be performed whenever available (Fig. 1). We found that immunostaining saved the pathologist’s time while searching for the microorganisms under the microscope due to the unambiguous features of the staining (personal observation), although Giemsa and Alcian blue stains also serve well as alternative stains with high sensitivity and specificity [24]. Patients with gastric MALT lymphoma positive for *H. pylori* have better responses to antibiotics eradication therapy. The persistence of *H. pylori* in gastric biopsies after antibiotics eradication therapy usually indicates reinfection or the development of bacteria species resistant to the antibiotics. Therefore, a change in the therapeutic protocol is recommended [25].

## Molecular cytogenetic findings of gastric MALT lymphoma

Many factors potentially affect the responsiveness of gastric MALT lymphoma to *H. pylori* eradication therapy. A balanced chromosomal translocation, t(11; 18)(q21;q21), results in a API2/MALT1 fusion gene, which is functional in activating the nuclear factor kappa B pathway and is associated with the pathogenesis of gastric MALT lymphoma. During translocation t(11;18)(q21;q21), the N-terminal region of API2 fuses to the C-terminal region of the MALT1 gene. Multiple breakpoints were identified in exons 7 and 8 of the API2 gene, and 3 major breakpoints were identified in the MALT1 gene. This cytogenetic abnormality could be demonstrated in approximately 20 % of gastric MALT lymphomas of Chinese patients, ranging from 14 to 22 % [26, 27]. The patients negative for translocation t(11; 18)(q21;q21) showed a higher response rate than those positive for the translocation (78 % versus 22 %); higher median survival time was also observed in the negative cases [18, 26]. Therefore, the status of t(11; 18)(q21;q21) with the lymphoma is a strong predictor for therapeutic response and patient prognosis. Interphase fluorescence in situ hybridization (FISH) may be performed using dual-color break-apart API2 and MALT1 probes or a dual-color dual-fusion probe. The test is highly sensitive and specific. Therefore, FISH for t(11; 18)(q21;q21) on formalin-fixed tissue sections should be performed routinely in newly diagnosed patients. Reverse transcriptase polymerase chain reaction (RT-PCR) was designed to detect the mRNA transcript of the API2/MALT1 fusion gene with high sensitivity and specificity. RT-PCR can be performed with tissue sections of paraffin-embedded, formalin-fixed tissue block. However, more tissue sections are required for the PCR reaction than for a FISH study. Other genetic abnormalities observed in gastric MALT lymphoma include translocations t(14;18)/IgH-MALT1, t(1;14)/BCL10-IgH and t(3;14)/FOXP1-IgH. However, these translocations are uncommon and their clinical significance has not been defined. Therefore, routine testing is not recommended [14].

**Fig. 2:**
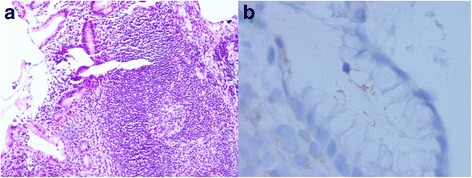
Gastric biopsy shows *H. pylori*-associated chronic active gastritis with scattered small lymphocytes and plasma cells; a lymphoid follicle with a reactive germinal center and a slightly expanded marginal zone (**a**, H&E, original magnification 100 X). Immunostaining of *H. pylori* shows intra-foveolar bacteria with curving configuration, characteristic of *H. pylori* (**b**, immunohistochemical stain of *H. pylori*, original magnificantion 1000 X)

The most common differential diagnosis of gastric MALT lymphoma is severe chronic gastritis. Careful morphologic examination of hematoxylin and eosin-stained biopsy tissue sections is essential for differential diagnosis. Chronic gastritis usually shows less dense lymphoid infiltrates with mixed small lymphocytes and plasma cells and a lack of lymphoepithelial lesions, and it also appears more polymorphic. Immunostaining for B-cell and T-cell markers, such as CD20, CD3 and CD43, is helpful in difficult cases and may reveal well-preserved B-cell and T-cell compartments without evidence of lymphoepithelial lesions (Fig. 2).

## Molecular diagnosis of gastric MALT lymphoma

In some cases, the histopathologic features are uncertain. Therefore, PCR for IgH gene rearrangement is helpful for a definitive diagnosis. Some diagnostic laboratories in China have adapted PCR protocols for B-cell receptor gene clonality analysis, which were originally developed by European Biomed-2 Study Group [28]. The PCR for IgH gene rearrangement covers a majority of B-cell lymphomas with high clinical sensitivity and specificity. Routine testing for kappa light chain gene rearrangement is not recommended. Multiple sets of PCR primers are designed to detect more than 90 % cases of B-cell lymphomas. Adequate positive and negative controls together with patient specimens must be performed each time. The results of the controls should be included in the diagnostic report of the PCR analysis. A result cannot be reported if the control sample does not work or the DNA is disqualified or suboptimal for the test. Clinical false-positive and false-negative results have been reported in approximately 5–10 % of confirmed B-cell lymphoma cases. Therefore, it is important to interpret the PCR results in combination with histopathologic and immunophenotypic findings. A more conservative approach to the diagnosis may be taken for those atypical cases to avoid overdiagnosis and intervention with aggressive therapy.

## Summary of pathologic diagnostic points of gastric MALT lymphoma

1. Infiltration of the gastric mucosa by dense, monomorphic small lymphocytes.
2. Lymphoepithelial lesions.
3. Dense B-cell infiltrates with sheet formation observed by immunohistochemical staining for CD20 and CD79a with aberrant expression of CD43.
4. *H. pylori* may be identified on hematoxylin and eosin-stained slides and confirmed by immunohistochemical staining.
5. In situ hybridization of the kappa and lambda light chains on tissue sections may show monoclonal light chain expression.
6. Monoclonal IgH gene rearrangement may be detected by PCR using formalin-fixed tissue and may be helpful in ambiguous cases.
7. API2/MALT1 gene rearrangement may be detected in approximately 20 % of the cases by FISH.

## Staging of gastric MALT lymphoma

As soon as a pathological diagnosis of gastric MALT lymphoma is established, clinical staging of the disease should be performed. The modified Ann Arbor (Musshoff) system is recommended for the staging (Table 2) [25]. The majority of cases with gastric MALT lymphoma are in the early stage of disease, with lymphoma limited to the gastric mucosa, submucosa (stage I1E) and less commonly, in the superficial layer of the muscularis propria (stage I2E). Endoscopic ultrasound for the detection of lymphoma invasion of the stomach wall has been used in European countries. Routine abdominal CT scan is recommended for possible regional gastric lymph node involvement (stage II1E). Bone marrow involvement is uncommon, and routine bone marrow biopsy for staging is not recommended for patients with negative regional lymphadenopathy.

**Table 2:** Modified Ann Arbor (Musshoff) staging [25]

Modified Ann Arbor system	Extension of organ involvement by lymphoma
I1E	Mucosa, submucosa involvement
I2E	Muscularis propria, serosa, neighboring organs
II1E	Regional abdominal lymph nodes (compartment I + II)
II2E	Intra-abdominal distant lymph nodes
IIIE	Extra-abdominal lymph nodes
IV	Diffuse or disseminated infiltration of distant or extra-gastrointestinal organs, bone marrow

## Management of gastric MALT lymphoma

Normal gastric mucosa lacks lymphoid aggregates; only rare scattered lymphocytes, plasma cells and eosinophils are identified in the gastric mucosa and submucosa. *H. pylori* infection triggers an inflammatory reaction with prominent neutrophil infiltration and lymphocytic proliferation. The lymphoid cells form lymphoid follicles with germinal center and minimal marginal zone expansion, which is characteristic for *H. pylori*-associated chronic active gastritis. The high prevalence of *H. pylori* infection and low incidence of gastric MALT lymphoma among the general population suggest that there is an interaction between *H. pylori* and host factors that determines the host susceptibility to MALT lymphoma. This observation has been supported by experimental studies. Certain genetic predispositions have been identified [29, 30]. An increased prevalence of both HLA-DQA1*0103 and HLA-DQB1*0601 alleles and DQA1*0103-DQB1*0601 haplotypes has been observed in patients with gastric MALT lymphoma [29]. Due to the close association of *H. pylori* with gastric MALT lymphoma, Wotherspoon, et al first used antibiotics to treat patients with gastric MALT lymphoma and induced dramatic regression of the lymphoma [6]. Since then, combined antibiotics for *H. pylori* eradiation has become a standard therapy for patients with gastric MALT lymphoma regardless of the status of *H. pylori* in the biopsy tissue. The majority of *H. pylori*-positive gastric MALT lymphomas are in early stage of disease, with involvement of the gastric mucosa and submucosa (stage I1E); invasion of the muscularis propria may be identified (stage I2E). Clinical studies have been performed for eradiation therapy of *H. pylori* gastritis and gastric MALT lymphoma over the last three decades. Guidelines for the diagnosis and management of *H. pylori* infection of the stomach were published and updated recently by the World Gastroenterology Organization, the American College of Gastroenterology, the Asian-Pacific Association of Gastroenterology and other professional organizations over the last decade (Table 3) [2, 7, 9, 11, 25, 31–36]. The guideline protocols are also adapted to *H. pylori* eradication therapy in gastric MALT lymphoma. A protocol combining a proton pump inhibitor (PPI) and two antibiotics, or “triple therapy,” has been recommended as first-line therapy until recently. However, recent studies have shown an increase in drug resistance to “triple therapy” in China and other areas of the world [4, 33, 37]. Protocols with 14-day therapy appeared to have a higher response rate than 7-day therapy without an increase in side effects [9, 36]. Sequential therapy showed an advantage over conventional “triple therapy” and was recommended for the replacement of conventional “triple therapy” as the first-line *H. pylori* eradication therapy [38]. However, this result was not confirmed by recent studies in China and other countries of the world [11, 39, 40].

**Table 3:** First-Line Regimens for *Helicobacter pylori* Eradication (ACG Guideline 2007) [7]

Regimen	Duration (days)	Eradication Rates	Comments
Triple therapy: ^∗^Standard dose PPI b.i.d. (esomeprazole is q.d.), clarithromycin 500 mg b.i.d., amoxicillin 1,000 mg b.i.d.	10–14	70–85 %	Consider in non-penicillin-allergic patients who have not previously received a macrolide
Triple therapy (penicillin allergic): Standard dose PPI b.i.d., clarithromycin 500 mg b.i.d., metronidazole 500 mg b.i.d.	10–14	70–85 %	Consider in penicillin-allergic patients who have not previously received a macrolide or are unable to tolerate bismuth quadruple therapy
Bismuth therapy: Bismuth subsalicylate 525 mg p.o. q.i.d. metronidazole 250 mg p.o. q.i.d., tetracycline 500 mg p.o. q.i.d., ranitidine 150 mg p.o. b.i.d. or standard dose PPI q.d. to b.i.d.	10–14	75–90 %	Consider in penicillin-allergic patients
Sequential therapy: PPI + amoxicillin 1 g b.i.d. ×5 followed by: PPI, clarithromycin 500 mg, tinidazole 500 mg b.i.d. ×5	10	*>*90 %	Requires validation in North America

Note: The above recommended treatments are not all FDA approved. The FDA-approved regimens are as follows:

Bismuth 525 mg q.i.d. + metronidazole 250 mg q.i.d. + tetracycline 500 mg q.i.d. × 2 weeks + H_2_RA as directed × 4 weeks

Lansoprazole 30 mg b.i.d. + clarithromycin 500 mg b.i.d. + amoxicillin 1 g b.i.d. × 10 days

Omeprazole 20 mg b.i.d. + clarithromycin 500 mg b.i.d. + amoxicillin 1 g b.i.d. × 10 days

Esomeprazole 40 mg q.d. + clarithromycin 500 mg b.i.d. + amoxicillin 1 g b.i.d. × 10 days

Rabeprazole 20 mg b.i.d. + clarithromycin 500 mg b.i.d. + amoxicillin 1 g b.i.d. × 7 days

*ACG* American College of Gastroenterology, *PPI* proton pump inhibitor, *p.o.* orally, *q.d.* daily, *b.i.d.* twice daily, *t.i.d.* three times daily, *q.i.d.* four times daily

^∗^ Standard dosages for PPIs are as follows: lansoprazole 30 mg p.o., omeprazole 20 mg p.o., pantoprazole 40 mg p.o., rabeprazole 20 mg p.o., esomeprazole 40 mg p.o

Although patients with gastric MALT lymphoma with no *H. pylori* are less responsive to *H. pylori* eradication, a portion of the *H. pylori*-negative cases are potentially curable by *H. pylori* eradication therapy alone [25]. The rationale for this finding has not been elucidated. It is suspected that some *H. pylori*-negative cases are false negatives due to patchy distribution of the microorganism in the gastric mucosa and limited tissue sampling during biopsy. PPI therapy before biopsy reduces the sensitivity of *H. pylori* detection. Therefore, PPI should be discontinued at least 2 weeks before *H. pylori* testing.

Follow-up examination was recommended in 3 months after finishing *H. pylori* eradication therapy. Tests such as the ^13^C urea breath test or the monoclonal stool antigen test should be considered together with gastric endoscopic examination and biopsy. The patient’s symptoms may disappear and *H. pylori* become undetectable with a negative breath test if the therapy is effective. The histological response usually lags behind the *H. pylori* eradication, and a lymphoma infiltrate may persist for 12 months or even longer. Scattered small lymphocytes and lymphoid aggregates may be identified with mild fibrosis (Fig. 2). The Groupe d’Etude des Lymphomes de l’Adult (GELA) grading system was recommended for the post-treatment evaluation of gastric MALT lymphoma (Table 4) [35, 38].

**Table 4:** GELA grading system for post-treatment evaluation of gastric MALT lymphoma [35]

GELA category	Histological features	Clinical category
Complete histological response (CR)	Total disappearance of lymphoid infiltrate with only scattered small lymphocytes and plasma cells. Regressive stromal changes with fibrosis and separation of glands can be observed.	Complete remission
Probable minimal residual disease (pMRD)	Small lymphoid aggregates present, stromal regressive changes are usually present.	Complete remission
Responding residual disease (rRD)	Overt residual lymphoma with a nodular or diffuse infiltrate of neoplastic B-cells but with clear evidence of regressive stromal changes characterized by fine fibrosis and an “empty lamina propria.”	Partial remission
No change (NC)	Persistence of overt lymphoma identical to that observed at diagnosis with no morphological features to suggest response to treatment (such as stromal fibrosis).	Stable or progressive disease

*GELA* Groupe d’Etude des Lymphomes de l’Adult, *MALT* mucosa-associated lymphoid tissue

Immunohistochemical staining for B-cell and T-cell markers is helpful for the examination. The subsequent follow-up examinations may be arranged every 6 months in the first year, and then the frequency may be reduced to every 12 months. CT-scan or PCR study for IgH gene rearrangement is generally not recommended during follow-up.

If the above first-line therapy fails to eradicate *H. pylori*, a second-line protocol should be considered. Increased numbers of strains of *H. pylori* resistant to clarithromycin and metronidazole have been reported during the last decade, ranging from 30 to 100 % [4, 33, 37]. “Quadruple therapy” including PPI, bismuth, tetracycline and metronidazole has been recommended [7]. The patient’s non-compliance may be one of the most common reasons for the failure of *H. pylori* eradication therapy. Discontinuation of medicine is common as soon as patients experience relief of symptoms or unexpected side effects. Therefore, patient education for therapy compliance is important. The question should be raised about therapeutic compliance if the first-line therapy fails before starting the second-line therapy. Testing for drug sensitivity is recommended when drug resistance is suspected. However, in clinical practice, a culture-based approach is unfeasible, especially in the developing world and China. Recently, point mutation analysis of *H. pylori* genes by PCR, such as the 23S rRNA and gyrA genes, has been used for assessing *H. Pylori* susceptibility to clarithromycin and levofloxacin, respectively [41, 42]. Other factors may affect the result of *H. pylori* eradication therapy and gastric MALT lymphoma. Non-steroidal anti-inflammatory drugs potentially affect the result of *H. pylori* eradication therapy. *H. pylori* eradication therapy should be performed after the discontinuation of non-steroidal anti-inflammatory drugs, according to the Japanese Ministry of Health, Labor and Welfare [43]. Tinidazole is an analogue of metronidazole and has been used in the sequential therapy protocol for *H. pylori* eradication. The inhibitory effect of tinidazole on parasites, such as amoebae and bacteria is similar to metronidazole. However, the side effects are more tolerable and last a shorter time. The most common side effects include nausea, bitter taste and itchiness. Drinking alcohol while taking tinidazole may cause vomiting, headache, shortness of breath, flushing and an increase in blood pressure. Therefore, alcohol should be avoided during therapy with tinidazole. Lower rates of drug resistance of *H. pylori* were found with tinidazole than with metronidazole in a recent in vitro study [2].

Primary gastric large B-cell lymphoma may be a de novo large B-cell lymphoma or may be transformed from gastric MALT lymphoma. Large B-cell lymphoma transformation from gastric MALT lymphoma is uncommon, ranging from 0.5 % to 1 % in a 7-year observation period [44, 45]. It is a more aggressive lymphoma, and systematic chemotherapy is recommended for the first-line treatment. However, the current antibiotics protocols for *H. pylori* showed limited side effects, and complete remission has been observed in a portion of the patients with gastric large B-cell lymphoma. Therefore, antibiotics therapy may be beneficial for those cases with early-stage gastric large B-cell lymphoma [16–19].

Radiation therapy is effective for patients with localized gastric MALT lymphoma and is recommended for patients who have early-stage disease and are refractory to antibiotics therapy. The dosage of radiation has been reduced over the last two decades. Wirth et al. reported a study comprising 102 patients with gastric MALT lymphoma, 58 previously untreated cases and 44 with recurrent or residual disease after prior medical or surgical therapy. The overall 5-year recurrence-free survival rate was 97 % with low doses of less than or equal to 30 cGy based on a median follow-up time of 7.5 years [46]. Another recent study included 22 patients with stage IE and IIE gastric MALT lymphoma, including 8 cases with t(11;18)(q21;q21) translocation. All patients reached complete remission after radiation with 30 cGy without severe toxicity. The overall and 5-year relapse-free survival rates were 91 and 84 %, respectively at a median follow-up time of 74 months [47, 48]. The side effects of radiation are usually transient and tolerable, including nausea, anorexia and vomiting. Low-dosage radiation therapy preserves the function of the stomach and avoids nutritional disease induced by surgical resection. Surgical intervention is preserved only for those patients with ulcers, acute bleeding and/or perforation.

Systematic chemotherapy should be considered only for those patients with advanced-stage disease with involvement of distant lymph nodes and/or bone marrow and those patients with large B-cell transformation. It was reported recently that 95 % of MALT lymphoma patients who failed in *H. pylori* eradication antibiotics therapy showed 95 % complete remission after combined chlorambucil and rituximab therapy, which was superior to single-drug chemotherapy with chlorambucil or rituximab alone [44, 45, 49–51]. R-CHOP [Rituximab, Cyclophosphamide, Hydroxydaunorubicin (adriamycin), Oncovin (vincristine) and Prednisone] is recommended as a standard protocol for gastric large B-cell lymphoma [52].

## Conclusions

*H. pylori* gastritis is one of the most common infectious diseases in China and worldwide. Gastric MALT lymphoma is a neoplasm associated with *H. pylori* infection and is the first malignant disease that can be cured by antibiotics therapy alone. The pathogenesis of gastric MALT lymphoma is not yet elucidated. The estimated incidence of gastric MALT lymphoma is low (0.3-0.4/100,000) in the United States and Europe, but the incidence is not known in China. The diagnosis of gastric MALT lymphoma can be challenging. Due to its rarity, most general pathologists may not be familiar with the diagnostic features of gastric MALT lymphoma. Therefore, underdiagnosis or overdiagnosis of this disease is not uncommon. Combined hematoxylin and eosin staining and immunohistochemical staining are routinely used for the pathologic diagnosis of the lymphoma. Special studies, such as molecular studies, are required to reach a definitive diagnosis in difficult cases. Expert consultation by hematopathologists may be beneficial for diagnosis. The *H. pylori* eradication regimen for early-stage gastric MALT lymphoma is similar to *H. pylori* gastritis. However, over-therapy, such as systemic chemotherapy for early-stage gastric MALT lymphoma, is not uncommon in the developing world, such as in China. *H. pylori* reinfection is common after eradication therapy due to cross-infection through person-to-person contact, especially among family members. Communication between gastroenterologists and oncologists is advised for the proper management of patients with gastric MALT lymphoma.

## Abbreviations

*H. pylori, Helicobacter pylori;* IgH, immunoglobulin heavy chain; MALT, mucosa-associated lymphoid tissue; PCR, polymerase chain reaction; PPI, proton pump inhibitor
